# Immune remodeling and atrial fibrillation

**DOI:** 10.3389/fphys.2022.927221

**Published:** 2022-07-22

**Authors:** Yajun Yao, Mei Yang, Dishiwen Liu, Qingyan Zhao

**Affiliations:** ^1^ Department of Cardiology, Renmin Hospital of Wuhan University, Wuhan, China; ^2^ Cardiovascular Research Institute of Wuhan University, Wuhan, China; ^3^ Hubei Key Laboratory of Cardiology, Wuhan, China

**Keywords:** atrial fibrillation, immune remodeling, atrial remodeling, immunomodulation, inflammation

## Abstract

Atrial fibrillation (AF) is a highly prevalent arrhythmia that causes high morbidity and mortality. However, the underlying mechanism of AF has not been fully elucidated. Recent research has suggested that, during AF, the immune system changes considerably and interacts with the environment and cells involved in the initiation and maintenance of AF. This may provide a new direction for research and therapeutic strategies for AF. In this review, we elaborate the concept of immune remodeling based on available data in AF. Then, we highlight the complex relationships between immune remodeling and atrial electrical, structural and neural remodeling while also pointing out some research gaps in these field. Finally, we discuss several potential immunomodulatory treatments for AF. Although the heterogeneity of existing evidence makes it ambiguous to extrapolate immunomodulatory treatments for AF into the clinical practice, immune remodeling is still an evolving concept in AF pathophysiology and further studies within this field are likely to provide effective therapies for AF.

## Introduction

Atrial fibrillation (AF) is the most common sustained arrhythmia in clinical practice and is associated with complications, such as heart failure and stroke (Lippi et al., 2021). In recent decades, the combination of “trigger” and “substrate” has been considered the major cause for the initiation and maintenance of AF. In atypical sites, including pulmonary vein ostia, coronary sinus, ligament of Marshall, abnormal automaticity or early and delayed afterdepolarizations induce ectopic activity and then initiate AF (Santangeli and Marchlinski, 2017). The substrate, which manifests as atrial remodeling, increases the likelihood of ectopic firing or re-entry. There are at least three main forms of remodeling: electrical remodeling, structural remodeling and autonomic neural remodeling. Electrical remodeling manifests as changes in the number and distribution of ion channels and gap junction proteins, leading to a shortened atrial effective refractory period (AERP) and increased AERP dispersion (Grunnet et al., 2012). Structural remodeling is characterized by progressive collagen deposition and atrial fibrosis, which is a consequence of cardiac fibroblast activation and extracellular matrix metabolic dysfunction (Sohns and Marrouche, 2020). Autonomic neural remodeling presents primarily as nonhomogeneous nerve sprouts and an imbalance in sympathetic and parasympathetic activity, which can be related to the release of nerve growth factor (NGF) (Shen et al., 2011). Interestingly, these mechanisms are not independent, and the development of AF arises from their mutual promotion and comprehensive effects (Wijesurendra and Casadei, 2019; Kusayama et al., 2021).

**FIGURE 1:**
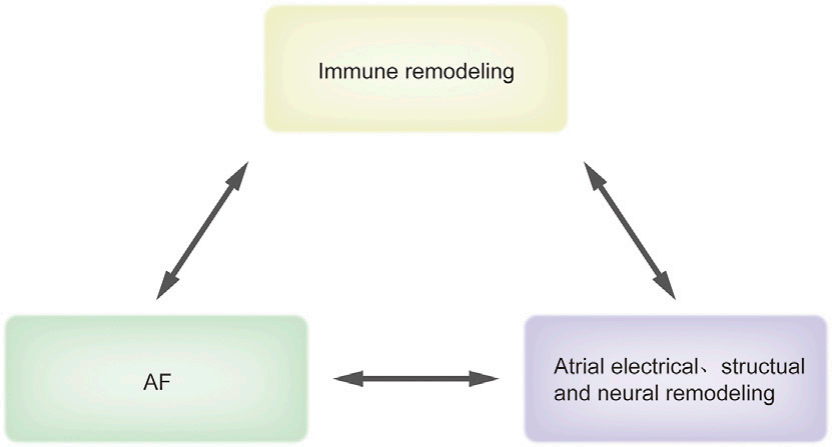
The association between immune remodeling and AF. This figure recapitulates briefly the association between immune remodeling and AF. Immune remodeling regulates atrial electrical, structual and neural remodeling to promote AF. In turn, AF-related pathologic changes including fibrosis can induce immune remodeling, which forms a positive circle loop. Abbreviations: AF, atrial fibrillation.

Furthermore, recent studies have shown that the immune system changes considerably and plays an essential role in the pathophysiological process of AF. Here, we regard the change of the immune system in AF as another form of remodeling and known as immune remodeling. It refers to the recruitment and activation of immune cells induced by various factors as well as the alternation in immune molecular secretion, which shapes a new immune network during AF (Li et al., 2021; Xiao et al., 2021). Moreover, immune remodeling goes throughout the whole process of AF development and maintenance (Miyosawa et al., 2020). On the one hand, immune remodeling regulates the known electrical, structural and neural remodeling to participate in the development of AF; on the other hand, the AF-related pathological changes including fibrosis act as a positive regulator of immune remodeling and further promote the maintenance of AF (Wernli et al., 2009; Rao et al., 2013; Fu et al., 2015) (Figure 1). Importantly, unlike atrial remodeling, immune remodeling is not limited to the atria and its effects on the peripheral circulation can not only facilitate clinical diagnosis but also show that AF should be considered as a systemic disease. However, the cause-effect-cause complexity makes it a bit more difficult to strictly distinguish between those events that might be the result of AF-induced immune remodeling or immune remodeling induced AF. Therefore, related immunology studies will greatly improve understanding of AF.

In this review, we focus on the association between immune remodeling and AF. First, we begin with a fundamental introduction to cardiac immunology components; then, based on clinical research data, the evidence of immune remodeling during AF is described in detail. Furthermore, we link immune remodeling to atrial electrical, structural and neural remodeling at the cellular and molecular levels. Finally, we summarize some potential therapies and aim to provide more precise and effective targets for AF treatment.

## Immune cells in cardiac homeostasis

Recent insights into the immune system and cardiology have suggested that immune cells are integral components for maintaining homeostasis in cardiac tissue (Hulsmans et al., 2017). The study on healthy adult mice showed that the immune cells constituted 4.7% ± 1.5% of the cardiac tissues. The frequencies of major immune cells in cardiac muscle were 12-fold higher relative to the skeletal muscles (Pinto et al., 2016; Ramos et al., 2017). Among them, approximately 81.4% ± 1.4% were myeloid cells, 8.9% ± 0.6% were B cells, 3.1% ± 0.4% were T cells and 6.6% ± 0.6% were non-myeloid/lymphoid immune cells (Pinto et al., 2016). However, this previous study is limited to ventricles without atria and valves. Another study on the adult human heart demonstrated the presence of 11 major cell types, including atrial cardiomyocytes, ventricular cardiomyocytes, fibroblasts, endothelial cells, and immune cells. Among them, myeloid and lymphoid immune cells accounted for 5.3% of the ventricular tissues and 10.4% of the atrial tissues (Litviňuková et al., 2020), which is in line with the findings in mice, which suggest that immune cells occupy a certain substantial proportion of the healthy heart. However, some differences may exist across species. For example, it was thought that the density of mast cells in the hearts of canines and humans was higher relative to the mouse heart (Gersch et al., 2002; Ingason et al., 2019). The distribution of immune cells in distinct cardiac areas is also non-homogeneous. Only a few studies have characterized the proportion of immune cell subsets in the atria. A precise understanding of immune cell subsets and their proportions in atria will pave the way for illustrating the mechanism underlying AF. In addition to the myocardium tissues, the pericardial fluid and adipose tissues contain some immune cells, which are important sources of tissue infiltration under stress (Butts et al., 2017; Horckmans et al., 2018). The origin, phenotypes, and functions of cardiac immune cells have been summarized in previous reviews (Lavine et al., 2018; Swirski and Nahrendorf, 2018; Varricchi et al., 2020).

## Immune remodeling during atrial fibrillation

### Evidence for immune remodeling

Immune remodeling is a multidimensional pathological process. The changes in the composition and number of immune cells can be regarded as morphologic or structural evidence for immune remodeling, while alternations in immune molecules can be considered as functional evidence of immune remodeling. In this review, we mainly summarize the current clinical evidence to support the concept of immune remodeling in AF.

Table 1 provides a summary of studies on the change in immune cell types in AF. CD45 is common to all inflammatory cells. As early as 2008, it was found that, as compared to that in individuals with normal sinus rhythm (SR), the infiltration of CD45^+^ cells was increased markedly in the atria of AF patients. Among those with AF, no difference in CD45^+^ inflammatory cell infiltration was observed between the left and right atria (Chen et al., 2008). A subsequent study by Yamashita and colleagues also supported this conclusion. They further demonstrated that in AF specimens, the infiltration of CD45^+^ and CD68^+^ cells in the atria endo- and sub-endomyocardium was predominantly high relative to the mid-myocardium and proposed that immune cells were recruited across the atrial endocardium during AF (Yamashita et al., 2010). Moreover, the number of peripheral CD45^+^ cells was increased significantly in patients with AF (Aguiar et al., 2019). Dendritic and mast cells have been observed in the atria of AF patients. The number of dendritic cells in AF patients was higher relative to those with SR, whereas the number of mast cells was similar (Smorodinova et al., 2017). Relative to the subjects with SR, the number of CD3^+^ T cells was increased significantly in the atrial tissue of patients with AF, which has been confirmed in several clinical studies (Smorodinova et al., 2017; Hohmann et al., 2020; Wu et al., 2020). However, whether the number of CD3^+^ T cells differs significantly in the AF subgroups remains controversial. A previous study argued that the number of CD3^+^ T cells increased from patients with SR to paroxysmal AF (pAF) and persistent AF (peAF), respectively. The number was lower in patients with permanent AF (permAF) relative to those with peAF (Hohmann et al., 2020). However, another study suggested that no statistical difference existed in CD3^+^ T cell infiltration between pAF and peAF/permAF (Wu et al., 2020). Differences in these findings may be due to the clinical heterogeneity of samples and the specificity of the antibodies. After all, these findings are highly dependent on the immunohistochemical and flow cytometry methods. Additionally, CD20^+^ B cells are occasionally present as small clusters in the epicardial layer and are very rare in the myocardium (Hohmann et al., 2020). As described previously, adipose tissue usually contains a far greater number of immune cells. The number of neutrophilic granulocytes and lymphocytes was higher in the atrial fat tissue of AF patients relative to SR individuals (Begieneman et al., 2015). Several animal studies have demonstrated the change in proportions of cardiac immune cells (especially macrophages) during the onset and maintenance of AF (Sun et al., 2016; He et al., 2021). In addition to routine inflammatory cells, activated platelets have also been proposed to be associated with AF. Toll-like receptors (TLRs) are expressed on the surface of platelets and participate in the platelet activation and thrombosis (Dib et al., 2020). A study found that compared with control group, peripheral and left atrial platelet TLR2 and TLR4 levels were significantly higher in AF patients. The above indicators were higher in atrium of peAF than that in pAF (Gurses et al., 2018). Another study in 2020 showed that increased platelet activation was found in peripheral blood from patients with hypertensive AF and the platelets were largely accumulated in these atriums (Liu et al., 2020), which implied the potential relation between platelets and AF. Taken together, the composition and number of immune cells are altered during AF.

**TABLE 1:** The main studies on the change of immune cells in AF.

Study	No. of patients	Sample source	Cell types	Detection methods
Chen et al. 2008	17 control, 18 AF	control:RAA AF:LAA, RAA	CD45^+^ cells	IHC
Yamashita et al. 2010	5 control, 11 AF	LA	CD45^+^ cells, CD68^+^ cells	IHC, IF
Liu et al. 2021	51 control, 50 pAF, 56 peAF	blood	CD3^+^ T cells	FLC
Begieneman et al. 2015	9 control, 33 AF	LAA (including fat tissue)	PMN, macrophages, lymphocytes	IHC
Smorodinova et al. 2017	27 control, 19 AF	LA, RA	CD3^+^cells, CD68KP1^+^ cells	IHC
Aguiar et al. 2019	9 control, 9 AF	blood	CD45^+^ cells and its subgroups	FLC
Hohmann et al. 2020	2 control, 2 pAF, 3 peAF, 3permAF	LAA	CD3^+^T cells, CD20^+^B cells	IF
Wu et al. 2020	14 control, 20pAF, 30peAF/permAF	LA (including fat tissue)	CD45^+^ cells, CD3^+^T cells	IHC
Liu et al. 2020	40 control, 25NAF, 25HAF	blood, atrium	platelet count, platelet activation	FLC, HC

Abbreviations: AF, atrial fibrillation; FLC, flow cytometry; HAF, hypertensive atrial fibrillation; IF, immunofluorescence; IHC, immunohistochemistry; LA, left atria; LAA, left atrial appendage; NAF, normotensive atrial fibrillation; pAF, paroxysmal AF; peAF, persistent AF; permAF, permanent AF; PMN, neutrophilic granulocyte; RA, right atria; RAA, right atrial appendage.

Functionally, inflammation is a primary, nonspecific response to the activation of innate and/or adaptive immunity. Several studies on the inflammation status of AF have been summarized in previous literature (Patel et al., 2010; Hu et al., 2015; Sagris et al., 2021). In this review, we highlight some direct evidence of the pathological alterations of immune molecules in AF (Table 2). In innate immunity, clinical studies showed that patients with AF had higher levels of circulating blood C-X-C motif ligand 1 (CXCL-1) and CXCL-12, which are critical regulators of monocyte/macrophage mobilization (Li et al., 2016; Zhang et al., 2020a). Galectin-3 is a β-galactoside binding lectin secreted by macrophages and its elevated levels can predict the progression from pAF to peAF (Wang Q et al., 2021). Macrophage migration inhibitory factor (MIF), a chemokine-like inflammatory cytokine, was also highly expressed in patients with AF, and AF progression corresponds to augmented MIF concentrations (Wan and Li, 2018). Additionally, as compared to the individuals without AF, atrial neutrophil extracellular traps and elevated serum myeloperoxidase (MPO) levels were frequent in patients with AF, which suggested infiltration and activation of neutrophils (Rudolph et al., 2010; Holzwirth et al., 2020). In adaptive immunity, it has previously been shown that patients with AF have higher levels of autoantibodies, including anti-β1-adrenergic receptor (anti-β1-AR) and anti-M2-muscarinic receptor (anti-M2-R) (Yalcin et al., 2015). Anti-M2-R can predict the degree of left atrial fibrosis in pAF patients and β1-AR autoantibody may promote the development of AF by regulating atrial fibrosis (Gurses et al., 2015; Shang et al., 2020). Moreover, the levels of circulating immunoglobulin-free light chains, kappa and lambda, were higher in individuals with AF (Matsumori et al., 2020). These alternations are inconsistent with the findings for rare B cells in the atria. This phenomenon can be explained by the fact that circulating antibodies but not local B cells are associated with AF development. A matched case-control study assessed the association of Th17-related cytokines [including interleukin-17A (IL-17A), IL-17F, IL-21, and IL-22] with AF and observed elevated plasma levels of Th17-related cytokines were independently related to high risk of AF (Wu et al., 2016). These molecules participate in the atrial electrical and structural remodeling and facilitate the development of AF substrates. Overall, the immune microenvironment, including immune cells and molecules, change both locally and systemically during AF.

**TABLE 2:** The main studies on the change of special immune molecules in AF.

Study	No. of patients	Sample source	Immune molecules	Detection methods
Rudolph et al. 2010	17 control, 10 pAF	LAA	MPO	IF, ELISA
Yalcin et al. 2015	75 control, 75 pAF	serum	anti-β1-R, anti-M2-R	ELISA
Gurses et al. 2015	31 control, 31 pAF	serum	anti-M2-R	ELISA
Li et al. 2016	20 control, 270 AF	serum	SDF-1α (CXCL12)	ELISA
Wu et al. 2016	336 control, 336 AF	serum	Th17 related cytokines	ELISA
Wan and Li. 2018	103 control, 66 pAF,68 peAF, 52 perm AF	serum	MIF	ELISA
Holzwirth et al. 2020	37 control, 121 AF	serum	MPO	ELISA
Zhang et al. 2020a	31 control, 31 AF	blood	CXCL1, CXCR2^+^ monocytes	ELISA, FLC
Matsumori et al. 2020	28 control, 28 AF	blood	immunoglobulin free light chains kappa and lambda	competitive-inhibition multiplex Luminex® assay

Abbreviations: AF, atrial fibrillation; anti-β1-R, anti-β1-adrenergic receptor; anti-M2-R, anti-M2-muscarinic receptor; CXCL, C-X-C motif ligand; CXCR, C-X-C motif receptor; ELISA, enzyme-linked immuno sorbent assay; FLC, flow cytometry; IF, immunofluorescence; LAA, left atrial appendage; MIF, macrophage migration inhibitory factor; MPO, myeloperoxidase; pAF, paroxysmal AF; peAF, persistent AF; permAF, permanent AF; SDF-1α, stromal cell-derived factor-1α.

### Mechanism of immune remodeling

The precise mechanisms of immune remodeling during AF are still elusive. On the one hand, it is possible that some risk factors and inflammation-related conditions, including hypertension, coronary atherosclerosis, obesity, sepsis and obstructive sleep apnea, can increase the release of damage-associated molecular patterns and/or pathogen-associated molecular patterns, leading to the activation of immune responses (Sirisinha, 2011). Cardiac surgical stimulation is also a potential trigger for immune cell activation, which explains the increased circulating immune cells and postoperative AF incidence after coronary artery bypass grafting (Hammer et al., 2021). Additionally, atrial rapid and irregular electrical activity causes intracellular calcium overload, oxidative stress and cell apoptosis, which in turn lead to more resident immune cell activation and cytokine release (Van Wagoner, 2008; Hu et al., 2015). This allows the formation of positive feedback loops between immune activation and AF. On the other hand, the function of negative regulators is impaired during AF, as shown by a lower number of Treg cells and limited function of anti-inflammatory mediators, including IL-4 and IL-10 (Sulzgruber et al., 2017). This point has been well proven by the finding that the depletion of spleen-derived IL-10 can augment AF vulnerability (Kondo et al., 2016). In addition, the programmed death-1(PD-1) and its ligand PD-L1 have recently been highlighted as critical regulators that maintain this immune balance by negatively regulating T cell activation, proliferation and cytokine production. The downregulation of the PD-1/PD-L1 signaling pathway in AF partially participates in AF pathogenesis (Liu et al., 2015). Moreover, the imbalance in autonomic nerve system (ANS) is associated with the development and maintenance of AF (Kusayama et al., 2021), which may be an important cause of immune remodeling.

## The role of immune remodeling in atrial fibrillation

### Immune remodeling and electrical remodeling

**FIGURE 2:**
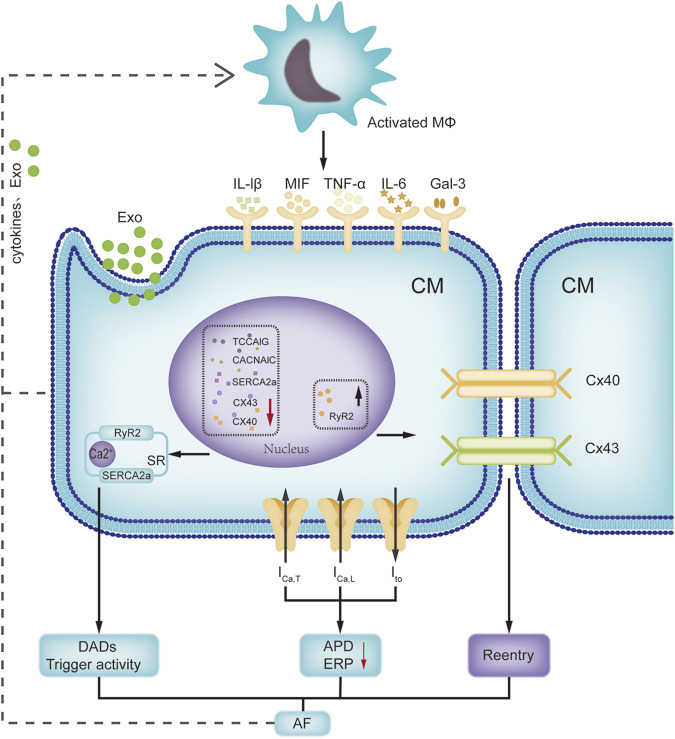
Interactions of immune remodeling and electrical remodeling. This figure focuses on the interactions between activated macrophage and cardiomyocyte. Activated macrophages can release cytokines and exosomes to affect the expression of ion channels and connexins in cardiomyocytes. Meanwhile, pacing cardiomyocytes can promote the macrophage activation. Abbreviations: APD, action potential duration; CACNA1C, L-type calcium channel α1C subunit; CM, cardiomyocyte; Cx 40, connexin 40; DAD, delayed after-depolarization; ERP, effective refractory period; Exo, exosomes; Gal-3, galectin 3; I_Ca-T_, T type calcium channel current; I_to_, transient outward potassium current; IL-6, interleukin-6; Mφ, macrophage; MIF, macrophage migration inhibitory factor; RyR2, ryanodine receptor 2; SR, sarcoplasmic reticulum; SERCA2a, sarcoplasmic reticulum Ca-ATPase; TCCA1G, T-type calcium channel α1G subunit.

Recent investigations have revealed that the immune system has an impact on atrial electrophysiology (Figure 2). With the activation of immune cells, a large amount of pro-inflammatory factors, including tumor necrosis factor-α (TNF-α), MIF, IL-1β, IL-6 and galectin-3 are released subsequently during AF, which can induce atrial electrical remodeling. Ca2^+^-associated abnormalities play a vital role in delayed afterdepolarizations and triggered activities (Schober et al., 2012). TNF-α can disrupt the intracellular calcium homeostasis in atrial myocytes by repressing the expression of T-type calcium channel α1G subunit (TCCA1G) and sarcoplasmic reticulum Ca-ATPases (SERCA2a) (Kao et al., 2010; Rao et al., 2016). TNF-α administration to the pulmonary vein cardiomyocytes affects multiple ionic currents (reduced *I*_*Ca-L*_ and increased *I*_*to*_), induces delayed afterdepolarizations, thereby enhancing arrhythmogenic activity (Lee et al., 2007). MIF treatment of HL-1 atrial myocytes increases calcium transients and sarcoplasmic reticulum calcium levels by inducing the expression of ryanodine receptor 2 (RyR2) (Cheng et al., 2020). In addition, activated immune cells such as macrophages prime the assembly of the NLRP3 inflammasome *via* TLR4 or nuclear factor-κB signaling, and trigger the release of IL-1β and IL-18. Macrophage-derived IL-1β can hinder quaking protein binding to the α1C subunit of L-type calcium channel (CACNA1C) and decrease calcium channel expression (Sun et al., 2016). Although macrophage NLRP3 activation is insufficient to cause AF, it has been shown that M1 macrophage-derived exosomes mediate cardiomyocyte NLRP3 activation by transferring miR-29a, while cardiomyocyte NLRP3 activation can upregulate the expression of RyR2 and promote abnormal sarcoplasmic reticulum Ca2^+^ release (Yao et al., 2018; Wang Y et al., 2021). Moreover, galectin-3 is also an important contributor for atrial electrical remodeling. Galectin-3-treated HL-1 myocytes have a shorter action potential duration, smaller *I*_*Ca-L*_ current, increased sarcoplasmic reticulum calcium content and ultrarapid delayed rectifier potassium current than control cells have. Specific neutralization of its membrane surface receptor CD98 significantly weakens galectin-3-induced Ca^2+^ handling imbalance (Cheng et al., 2022). Consistent with these results, Galectin-3 inhibitor GMCT treatment could mitigate pacing-induced electrical remodeling and abnormal Ca^2+^ handling in a sheep model (Takemoto et al., 2016).

The altered expression and distribution of connexins (Cx) on atrial myocytes disable gap junctional intercellular communication and reduce conduction velocity in the atrium, leading to increased vulnerability to AF (Nagibin et al., 2016). Mouse recombinant MIF can concentration-dependently downregulate Cx43 expression in atrial myocytes by activating ERK1/2 kinase (Li et al., 2017). A recent study verified that elevated IL-6 levels rapidly lowered the expression of cardiac Cx43 and Cx40 (Lazzerini et al., 2019). In addition, TNF-α has the ability to change the expression and distribution of Cx43 and Cx40 (Liew et al., 2013). In an Ang II-infused hypertensive mouse model, Cx43 delocalization was obvious, while adoptive transfer of Treg cells induced normal Cx43 localization at the intercalated disk regions (Kvakan et al., 2009), which indicated that a reduced proportion of Treg cells in patients with AF might promote electrical remodeling by controlling Cx43 (Sulzgruber et al., 2017). Overall, immune cell-mediated electrophysiology and inflammatory response promote atrial electrical remodeling in an indirect or direct manner. Moreover, tachypacing of HL-1 atrial myocytes or Ang II-treated atrial myocyte-derived exosomes can promote M1 macrophage polarization (Sun et al., 2016; Cao et al., 2021). There is a reciprocal interaction between immune remodeling and electrical remodeling. There is no doubt that deep and systematic research on immune-electrophysiology will provide new perspectives for AF treatments.

### Immune remodeling and structural remodeling

**FIGURE 3:**
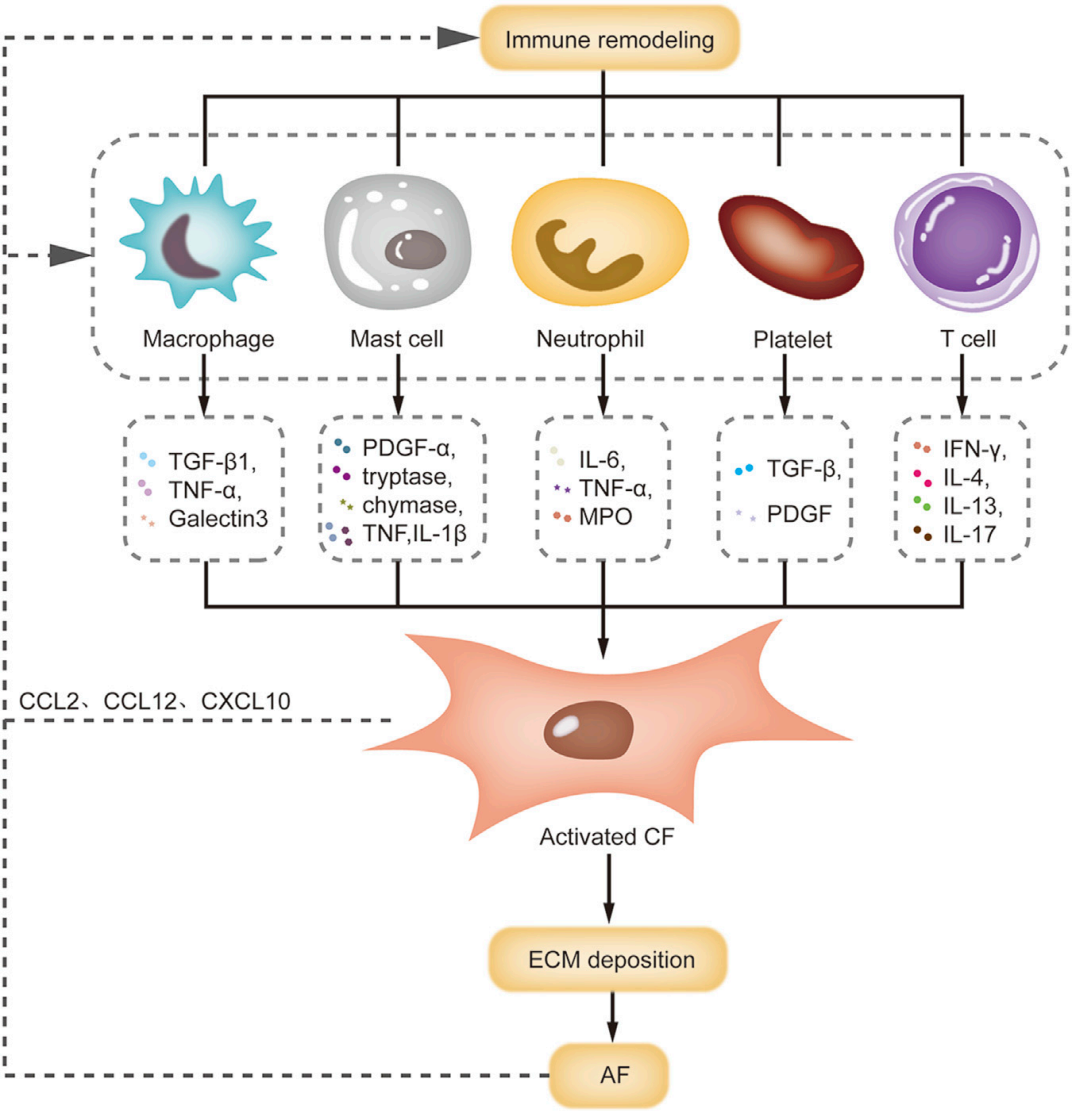
Interactions of immune remodeling and structural remodeling. This figure summarizes the interactions between immune cells and CF during AF. Macrophage, mast cell, neutrophil and T cell can release pro-fibrotic factors to activate CF, leading to ECM deposition and atrial structural remodeling. Meanwhile, activated CF can promote the macrophage recruitment by releasing chemokines. Abbreviations: CCL, C-C motif ligand; CF, cardiac fibroblast; CXCL, C-X-C motif ligand; ECM, extracellular matrix; IFN-γ, interferon-γ; IL-4, interleukin-4; MPO, myeloperoxidase; PDGF, platelet-derived growth factor; TGF-β, transforming growth factor-β; TNF-α, tumor necrosis factor-α.

Numerous studies have indicated that the infiltration of immune cells participates in atrial fibrosis and this effect depends mainly on the secretion of the cytokines (Figure 3). Macrophages, as major sources of transforming growth factor-β1 (TGF-β1) during the fibrotic process, can induce fibroblast-to-myofibroblast differentiation (Fadok et al., 1998). TNF-α is also involved in the pathogenesis of atrial fibrosis through activation of the TGF-β signaling pathway and increased secretion of matrix metalloproteinases (MMPs) (Liew et al., 2013). Galectin-3 produced by macrophages interacts with TGF-β and induces atrial fibrosis by stimulating the downstream TGF-β1/Smad pathway (Xiao et al., 2020). In addition, various chemokine receptors, including C-X-C chemokine receptor 2 (CXCR2), CXCR4, and CXCR6, are expressed on monocytes/macrophages and involved in atrial fibrosis by mobilizing macrophages (Zhang et al., 2020a; Liu et al., 2021). It has been demonstrated that Ang II can induce chemokine expression in atrial fibroblasts, thereby inducing the chemotaxis of macrophages (Chen et al., 2015). There seems to be a positive feedback loop between macrophages and fibrosis, which is pivotal in the development of AF substrate.

Mast cells are involved in atrial structural remodeling. In a mouse model of diabetes, hyperglycemia led to mast cell infiltration in the atria, atrial fibrosis and increased AF susceptibility. Transgenic mast-cell deficiency reversed these pathological changes (Uemura et al., 2016). In a pressure-overload mouse model, activated mast cells promoted fibroblast activation and fibrosis by releasing platelet-derived growth factor α (PDGFα). Neutralizing the PDGFα receptor with a specific antibody alleviated atrial fibrosis and AF susceptibility (Liao et al., 2010). Activated mast cells can also degranulate to produce some preformed mediators, including histamine, tryptase and chymase, which can mediate the progression of atrial fibrosis (Patella et al., 1995). Mast cells also serve as sources of TNF and IL-1β, promote fibrotic remodeling by inducing inflammation and MMP9 production (Mukai et al., 2018). However, due to some anti-fibrotic mediators in mast cells, the role of mast cells in fibrosis is controversial.

Neutrophils release cytokines such as IL-6, TNF-α and MPO to accelerate atrial fibrosis. In Ang II-treated mice, neutrophil infiltration was accompanied by profoundly enhanced atrial fibrosis and elevated susceptibility to AF. MPO knockout blunted atrial fibrosis and protected Ang II-treated mice from AF by modulating MMP activity and hypochlorous acid formation (Rudolph et al., 2010). In addition, another study showed that the integrin CD11b/CD18 mediate neutrophil infiltration and localization within the atrial tissue (Friedrichs et al., 2014), which provides a potential novel avenue of treatment in AF. Platelets are activated in patients with AF. Once activated, platelets can release a large number of pro-fibrotic cytokines and grow factors (including TGF-β1 and PDGF) into blood and local tissue (Mussano et al., 2016; Karolczak and Watala, 2021). In Ang II-infused mice model, both clopidogrel treatment and platelet-specific deletion of TGF-β1 reduced Ang II-induced atrial fibrosis and AF induction (Liu et al., 2020).

Multiple studies have also shown that T cell infiltration plays a functional role in cardiac fibrosis. This role is highly dependent on cardiac milieus and T cell subsets. In a myocardial infarction model, Th1 cells appeared to exert an anti-fibrotic effect by secreting interferon-γ. Conversely, Th2 cells counteracted to the Th1 response by secreting several pro-fibrotic cytokines (IL-4 and IL-13) (Bradshaw and DeLeon-Pennell, 2020). Lu et al. (2020) demonstrated that increased Th17 cells and decreased Treg cells aggravated myocardial fibrosis by activating the IL-17/ERK1/2-AP-1 pathway. This trend was also observed in immune remodeling in AF (He et al., 2018). However, the biological role of these subtypes has not been established in AF.

### Immune remodeling and neural remodeling

The cardiac ANS plays a significant role in the occurrence and maintenance of AF. Recent studies have suggested that the immune system and ANS are intimately linked. Next, we will conclude the interactions between the immune system and ANS, and propose a number of blind spots in the existing experimental research on AF.

During immune remodeling, activated macrophages, mast cells and T cells can regulate neural remodeling by releasing inflammatory factors including NGF, IL-1β and IL-17A (Chaldakov et al., 2014; Lyu et al., 2020). An experimental study showed that catecholamine-induced inflammatory environment could promote the release of NGF from macrophages and regulate cardiac sympathetic remodeling (Lyu et al., 2020).The activation of NLRP3 inflammasome in macrophages can’t only ignite inflammatory reaction but also facilitate the sympathetic innervation (Yin et al., 2017; Lee et al., 2021). The key role of macrophages in the regulation of neural remodeling has also been tested *in vivo* by clodronate liposomes-mediated macrophage depletion. It was revealed quite early that intravenous clodronate liposomes could decrease cardiac sympathetic axon density and NGF expression following myocardial infarction (Wernli et al., 2009). Local macrophage depletion in stellate ganglia could also restrain cardiac sympathetic sprouting and ventricular arrhythmias in heart failure (Zhang D et al., 2021). A recent study showed that clodronate liposome injection into the atria in canines significantly decreased AF vulnerability after acute stroke, but nerve density and NGF expression were not assessed (Wang et al., 2019). In addition, exogenetic IL-1β or IL-17A microinjection into the left stellate ganglion (LSG) promoted neuronal remodeling of the LSG and deteriorated ventricular electrophysiology by regulating the neural inflammation, which were improved by a specific neutralizing antibody (Wang et al., 2017; Deng et al., 2019). The LSG also plays an important role in the initiation and maintenance of AF. However, atrial electrophysiology parameters were not measured at the same time. Although there is few direct evidence on immune-mediated neural remodeling in AF, these findings indicate that it may be a novel vantage point for understanding AF mechanism.

The autonomic activity also plays a vital role in immune remodeling. Sympathetic nervous system activation can regulate immune system function through β-ARs, which exist in almost all immune cell types. β1-AR is primarily expressed in innate immune cells, where its activation can increase LPS-induced production of inflammatory mediators (Speidl et al., 2004). β2-AR is the most highly and widely expressed β-AR isoform in immune cells, and its effect is highly dependent on the initiation of downstream signaling (Yoshida et al., 2015; Grisanti et al., 2016). β3-AR has also been shown to be significant in mediating immune cell mobilization and egress from the bone marrow (Méndez-Ferrer et al., 2010). Additionally, some non-immune cells expressing β-AR also involve in ANS-mediated immune remodeling. Renal collecting duct epithelial cells express β2-AR and play a key role in the heart-brain-kidney network. When sympathetic nervous system activation stimulates the KLF5-S100A8-S100A9 pathway in collecting duct epithelial cells, renal macrophages produce TNF-α, which in turn stimulates renal endothelial cells to secrete colony stimulating factor 2 into the circulation, thereby activating cardiac resident macrophages (Fujiu et al., 2017). These results suggest that sympathetic neural activation can not only directly regulate cardiac immune cell activation but also indirectly modulates myelopoiesis and immune cell mobilization to the heart by affecting other organs, such as the bone marrow and kidney. SNS activation in AF is well known, along with migration and infiltration of immune cells and the inflammatory cascade. It was demonstrated that renal sympathetic denervation in canines could suppress AF and reduce the increasing trend of TNF-α and IL-6 induced by rapid atrial pacing (Wang et al., 2013). Acute middle cerebral artery occlusion in canines led to an increase in LSG activity, atrial β1-AR expression, atrial macrophage infiltration and AF vulnerability, while ablation of the LSG reversed these changes (Wang et al., 2019; Yang et al., 2020). Although the definitive mechanism is not very clear, it is possible that the effect is related to sympathetic nerve-regulated immune remodeling.

The parasympathetic nervous system regulates immune system function through nicotinic and muscarinic acetylcholine receptors (nAChRs and mAChRs) in most immune cells. The α7 subunit of nAChR (α7nAChR) is the most studied and involved in cholinergic anti-inflammatory pathway. Research of canines with rapid atrial pacing uncovered that low-level vagus nerve stimulation (LL-VNS) significantly suppressed atrial electrical remodeling and AF inducibility, accompanied by low levels of TNF-α and IL-6 in the left atria (Zhang S. J et al., 2021). Spinal cord stimulation facilitated the effect of VNS and reduced the induction of AF (Dai et al., 2017). Researchers also further demonstrated that median nerve stimulation could heighten cardiac vagal tone and atrial ACh levels, and reverse the enhanced inflammation response and AF inducibility by short-term rapid atrial pacing (Zhao et al., 2018). These results are associated with inflammatory macrophage inhibition mediated by the cholinergic anti-inflammatory pathway. Despite suboptimal evidence, it could be speculated that the autonomic activity can regulate immune remodeling in AF.

The ANS activity is identified as a major component of the emotion response. Previous studies suggested that negative emotion including sadness, depression, anger and stress could increase the likelihood of symptomatic AF (Lampert et al., 2014). Depression is independently associated with AF recurrence after catheter ablation (Zhuo et al., 2020). The exact mechanisms are still mysterious. Recently, accumulating evidence have indicated the existence of immune remodeling during depression (Wittenberg et al., 2020). In major depressive disorder patients, anxious distress was highly associated with innate immune activation including higher levels of IL-6, TNF-α, monocyte chemoattractant protein-1 and increased number of activated monocytes in circulation (Gaspersz et al., 2017; Nowak et al., 2019). This shows that immune remodeling can be one of mediators of emotion-induced AF. In another study, major depressive disorder-derived monocytes displayed higher proportion of M1 polarization phenotype under standard culture conditions, but, higher M2 polarization when co-stimulated with autologous sera (Cosma et al., 2021). This fully reflects the plasticity of immune cells and potential value of immune regulation. However, the understanding for emotion-immune-AF correlation still be not thorough and deeper research is warrant.

## Therapeutic potential of targeting immune remodeling

The mechanisms underlying AF have been studied in the last several decades. Although a wide range of modalities contributes to the management of AF, the treatment efficacy in patients with AF remains suboptimal. Immune remodeling induces inflammation and is highly associated with atrial electrical, structural, and neural remodeling. Despite a scarcity of studies on interventions for immune remodeling, therapies targeting immune remodeling are promising.

In this section, we will draw from data, following five dimensions to briefly summarize the therapeutic potential of immunomodulation: 1) regular exercise; 2) anti-inflammatory therapy; 3) inflammatory cytokines-targeted therapy; 4) immune cells-targeted therapy, and 5) upstream regulation of immune remodeling.

Exercise has undeniable impacts on the immune (Duggal et al., 2019) and cardiovascular systems (Meissner et al., 2011). Regular physical exercise at low-moderate intensity is recommended as a feasible non-pharmacological therapy for AF patients (Chung et al., 2020). However, the underlying mechanistic detail remains unclear but can be attributed to the modulation of immune, fibrosis, and vagal tones (Guasch et al., 2013; Aschar-Sobbi et al., 2015). It has been suggested that regular voluntary physical activity alters the proliferation of hematopoietic stem and progenitor cells *via* modulation of their niche and reduces the inflammatory leukocyte output (Frodermann et al., 2019). Monocytes and neutrophils are extremely important elements for atrial inflammation and other risk factors for AF. However, a study suggested that exercise might have a dichotomous effect on the immune system in populations carrying a high burden of AF risk factors. High-intensity and long-term physical training resulted in increased leukocyte output and pro-inflammatory cytokine (TNF-α, IL-2, IL-6 and IL-8) release (Santos et al., 2007; Kawanishi et al., 2015; de Barcellos et al., 2021), along with a greater susceptibility to AF (Guasch et al., 2013; Aschar-Sobbi et al., 2015). Recently, Valenzuela and his colleagues performed a meta-analysis with 6 (*n* = 935,742) and 4 (*n* = 2,422) studies to analyze the association of AF with physical activity or sports practice, respectively. Their results suggested that physical activity was overall inversely associated with incident AF whereas high-intensity physical training in athletes was associated with a higher risk for AF (Valenzuela et al., 2022). Of note, there is not yet a strong evidence expounding the association of sports with AF.

Inflammatory responses are the most evident feature of immune remodeling. The widely available, low-cost, and anti-inflammatory drug, colchicine, was once thought to be have preventive potential for postoperative AF. In a sub-study of the COPPS trial, colchicine 1 mg twice daily on the third postoperative day, followed by 0.5 mg twice daily for a month, lowered the incidence of postoperative AF at 30 days compared to placebo (Imazio et al., 2011). Deftereos et al. also showed that colchicine administration, at a dose of 0.5 mg twice daily, for 3 months after pulmonary vein isolation in patients with paroxysmal AF, resulted in a significantly lower rate of AF recurrence over a median of 15 months of follow-up (Deftereos et al., 2014). However, a recent randomized controlled trial (1 mg of colchicine 24 h before the surgery, as well as on days 2, 3, 4, and 5 in the postoperative period) did not detect any statistically significant differences between the control and colchicine groups within 7 days after surgery (Shvartz et al., 2022). Two other studies also found similar results (Bessissow et al., 2018; Tabbalat et al., 2020). Although the above-mentioned opposite outcomes may be attributed to the small sample size and early study termination, the effectiveness of colchicine in preventing AF needs to re-examination. The release of cytokines/-chemokines is an important modulation of the immune system. Targeted treatment of specific cytokines or receptors has attracted considerable academic interest. In rats with sterile pericarditis, treatment with anti-IL-17A monoclonal antibodies markedly alleviated inflammation and fibrosis and suppressed the development of AF (Fu et al., 2015). Zhang et al. (2020b) also showed that targeting the CXCL-1/CXCR2 signaling could prevent and reverse the development of AF in spontaneously hypertensive rats. Trials in this research direction have already been conducted in clinical settings. A large, randomized clinical trial involving 10,061 patients with previous myocardial infarction demonstrated that canakinumab targeting of IL-1β significantly lowered the risk of recurrent cardiovascular events as compared to placebo (Ridker et al., 2017). Another trial in patients with peAF showed that canakinumab administration after electrical cardioversion could lower the incidence of AF recurrence at six months; however, no significant differences were observed due to the limited sample size (Krisai et al., 2020). Thus, the effectiveness of these drugs warrants further evaluation in larger multicenter randomized clinical trials.

Immune cells constitute the main factors for immune remodeling in AF. Targeted cell therapy may provide an effective strategy. Sun et al. (2016) suggested that the depletion of macrophages can relieve LPS-induced atrial electrical remodeling and AF vulnerability in mice. Wang et al. (2019) also confirmed the effects of depleting macrophages on AF induction. However, prior studies have showed that macrophages played an indispensable role in cardiac homeostasis. Thus, this approach remains controversial (Hulsmans et al., 2017). In addition, a recent study employed engineered CD8^+^ T cells as a therapeutic agent for treating cardiac fibrosis. Adoptively transferred antigen-special CD8^+^ T cells could target the fibroblast activation protein expressed on cardiac fibroblasts, thereby suppressing cardiac fibrosis (Aghajanian et al., 2019). This approach can be attempted in future investigations on AF treatment. Finally, immune remodeling may be modulated by a complex combination of physical and neuro-humoral factors in AF. LSG ablation has been shown to reduce macrophage infiltration in the atria and vulnerability to AF after an acute stroke (Wang et al., 2019). Therefore, treatment regimens targeting the upstream modulators of immune remodeling can be very efficient. However, the majority of the data derived from animal experiments. Due to differences among species in several aspects, especially the immune system, there is still a long way to verifying the efficacy and achieving the translation from animal experiments to clinical settings.

## Conclusion and future prospects

During AF, changes in the composition and number of immune cells, as well as in the levels of immune molecules, constitute immune remodeling, which is inextricably linked with atrial electrical, structural and neural remodeling. Recent attempts to prevent AF by modulating immune remodeling have also suggested the important role of immunity in AF. However, the complexity of immune cell subtypes and the heterogeneity of existing research also make the translation from current data to clinical practice both promising and challenging. Overall, this area of research is only beginning to evolve, and in-depth studies are still needed.
